# Analysis of asymmetry in lipid and content mixing assays with reconstituted proteoliposomes containing the neuronal SNAREs

**DOI:** 10.1038/s41598-020-59740-7

**Published:** 2020-02-19

**Authors:** Yun-Zu Pan, Xiaoxia Liu, Josep Rizo

**Affiliations:** 1 Department of Biophysics, University of Texas Southwestern Medical Center, Dallas, Texas United States; 2 Department of Biochemistry, University of Texas Southwestern Medical Center, Dallas, Texas United States; 3 Department of Pharmacology, University of Texas Southwestern Medical Center, Dallas, Texas United States; 4 Key Laboratory of Cell Differentiation and Apoptosis of Chinese Ministry of Education, Department of Pathophysiology, Shanghai Jiao Tong University School of Medicine, Shanghai, China

**Keywords:** Exocytosis, Membrane biophysics

## Abstract

Reconstitution assays with proteoliposomes provide a powerful tool to elucidate the mechanism of neurotransmitter release, but it is important to understand how these assays report on membrane fusion, and recent studies with yeast vacuolar SNAREs uncovered asymmetry in the results of lipid mixing assays. We have investigated whether such asymmetry also occurs in reconstitution assays with the neuronal SNAREs, using syntaxin-1-SNAP-25-containing liposomes and liposomes containing synaptobrevin (T and V liposomes, respectively), and fluorescent probes to monitor lipid and content mixing simultaneously. Switching the fluorescent probes placed on the T and V liposomes, we observed a striking asymmetry in both lipid and content mixing stimulated by a fragment spanning the two C_2_ domains of synaptotagmin-1, or by a peptide that spans the C-terminal half of the synaptobrevin SNARE motif. However, no such asymmetry was observed in assays performed in the presence of Munc18-1, Munc13-1, NSF and αSNAP, which coordinate the assembly-disassembly cycle of neuronal SNARE complexes. Our results show that switching fluorescent probes between the two types of liposomes provides a useful approach to better understand the reactions that occur between liposomes and detect heterogenous behavior in these reactions.

## Introduction

The release of neurotransmitters by Ca^2+^-triggered synaptic vesicle exocytosis is an exquisitely regulated process that is critical for communication between neurons. Release occurs in a series of steps that include tethering of synaptic vesicles to specialized areas of the presynaptic plasma membrane called active zones, priming of the vesicles to a release-ready state, and fusion of the vesicle and plasma membranes when an action potential triggers Ca^2+^ influx into the presynaptic terminal^1^. Extensive studies using varied approaches during the past three decades have yielded crucial insights into the sophisticated protein machinery that controls release^2–6^ and have allowed reconstitution of basic aspects of synaptic vesicle fusion with the eight most central components of this machinery^7,8^, leading to defined models for their functions. The neuronal SNAP receptors (SNAREs) synaptobrevin, syntaxin-1 and SNAP-25 play a central role in membrane fusion by forming a tight SNARE complex that brings the synaptic vesicle and plasma membranes together^9–12^. N-ethylmaleimide sensitive fusion protein (NSF) and soluble NSF adaptor proteins (SNAPs) disassemble SNARE complexes to recycle the SNAREs for another round of fusion^9,13,14^, whereas Munc18-1 and Munc13-1 mediate SNARE complex assembly in an NSF-SNAP-resistant manner^7,15^. The underlying mechanism involves binding of Munc18-1 to a self-inhibited ‘closed’ conformation of syntaxin-1^16,17^ and to synaptobrevin, thus templating SNARE complex assembly^18–21^ while Munc13-1 bridges the two membranes^8,22^ and facilitates opening of syntaxin-1^23–25^. This pathway of SNARE complex assembly is believed to enable regulation of release through a wide variety of mechanisms that depend on Munc13-1^2,20,26^ and is likely selected over other potential SNARE complex assembly pathways through inhibition of these pathways by αSNAP^27^. Fast, synchronous neurotransmitter release is triggered by the Ca^2+^ sensor Synaptotagmin-1 via interactions with the membranes and the SNARE complex^28–31^.

Despite these and other advances, fundamental questions remain about how the functions of the components of the release apparatus are integrated to trigger fast, Ca^2+^-dependent membrane fusion and how neurotransmitter release is regulated during diverse presynaptic plasticity process that underlie distinct forms of information processing in the brain^2^. Reconstitution assays designed to investigate membrane fusion between proteoliposomes using fluorescent probes have played a prominent role in dissecting the mechanism of release^2,6^ and are expected to be crucial to address these outstanding questions. Thus, it is important to understand in detail the nature of these assays and how they inform on membrane fusion. For instance, early pioneering studies using reconstituted neuronal SNAREs alone or together with a soluble synaptotagmin-1 fragment (e.g.^32,33^) were insightful but relied mostly on lipid mixing assays, and it is well established that evidence of lumenal content mixing without leakiness is crucial to demonstrate true membrane fusion^2,6,34–36^, as extensive lipid mixing between liposomes can occur with little content mixing (e.g.^37^). In this context, studies of yeast vacuolar fusion led to the observation of a striking asymmetry in lipid mixing assays: whereas lipid mixing was observed when liposomes containing fluorescent lipids and the synaptobrevin homologue Nyv1 were mixed with liposomes containing the homologues of syntaxin-1 and SNAP-25 (Vam3, Vti1 and Vam7), much less lipid mixing was observed when the fluorescently labeled lipids were included instead in the Vam3-Vti1-Vam7-liposomes^38^. These results indicated that the lipid mixing observed in the former configuration could not arise from liposome fusion or even from hemifusion.

The study presented here was designed to test whether asymmetry in lipid mixing can also occur in reconstitution experiments performed with proteoliposomes containing neuronal SNAREs. For this purpose, we used an assay that simultaneously monitors lipid and content mixing, and switched the dyes placed on the different types of liposomes. We observed clear asymmetry in lipid mixing mediated by the neuronal SNAREs alone or together with a soluble synaptotagmin-1 fragment in the presence of Ca^2+^. Surprisingly, we also observed asymmetry in context mixing under these conditions. In contrast, minimal asymmetry in both lipid and context mixing was observed in reconstitution assays that incorporated Munc18-1, a Munc13-1 fragment, NSF and αSNAP in addition to the neuronal SNAREs. These results emphasize the importance of monitoring multiple parameters to characterize reactions between liposomes and show that switching the fluorescent dyes between the two types of liposomes provides an avenue to better understand these reactions.

## Results

**Figure 1:**
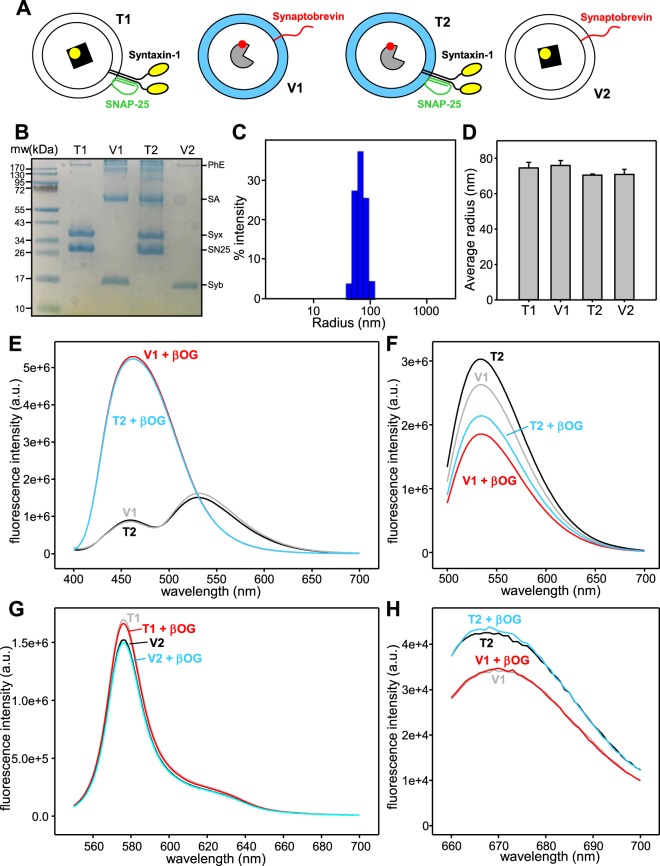
Characterization of proteopliposomes. (**A**) Diagrams illustrating the compositions of T1, V1, T2 and V2 liposomes. The light blue color in the membranes of the V1 and T2 liposomes represents the mixture of fluorescent lipids (NBD-PE and MB-PE). The PhycoE-Biotin trapped in the lumen of the T1 and V2 liposomes is represented by a black square with a yellow circle, whereas the Cy5-SA trapped in the lumen of the V1 and T2 liposomes is represented by a pie shape with a red circle. Note that syntaxin-1 and SNAP-25 are shown forming a heterodimer with a 2:1 stoichiometry (reviewed in^52^). (**B**) Equal amounts of T1, V1, T2 and V2 liposomes were analyzed by SDS-PAGE and coomassie blue staining. The positions of molecular weight markers are indicated on the left, and those of synaptobrevin (Syb), SNAP-25 (SN25), syntaxin-1 (Syx), SA and PhycoE (PhE) are indicated on the right. The uncropped gel is shown in Supporting Fig. S1. (**C**) DLS analysis of a sample of T2 liposomes illustrating the particle size distribution typical of the liposome preparations. (**D**) Average radii of representative samples of T1, V1, T2 and V2 liposomes obtained from three independent determinations by DLS. Error bars indicate standard deviations. (**E**) Fluorescence emission spectra of V1 and T2 liposomes before and after addition of β-OG. The MB fluorescence was excited at 370 nm. (**F**) Fluorescence emission spectra of the same liposomes as in panel (**E**), exciting the NBD fluorescence at 465 nm. (**G**) Fluorescence emission spectra of T1 and V2 lipososomes before and after addition of β-OG. The PhycoE fluorescence was excited at 488 nm. (**H**) Fluorescence emission spectra of V1 and T2 liposomes before and after addition of β-OG. The Cy5 fluorescence was excited at 645 nm.

The approach that we use to simultaneously monitor lipid and content mixing between liposomes containing synaptobrevin (V) and liposomes containing syntaxin-1 and SNAP-25 (T-liposomes)^8,39^ was originally developed in studies of yeast vacuolar fusion^38,40^ and involves incorporation of marina blue (MB)- and 7-nitrobenz-2-oxa-1,3-diazole (NBD)-labeled lipids in the V-liposomes, as well as Cy5-streptavidin (Cy5-SA) and biotinylated R-Phycoerythrin E (PhycoE-biotin) in the V- and T-liposomes, respectively. MB and NBD form a donor-acceptor pair for fluorescence resonance energy transfer (FRET), and increase in the MB emission fluorescence intensity due to loss of FRET with NBD upon lipid dilution reports on lipid mixing. PhycoE and Cy5 provide another FRET donor-acceptor pair, and increase in the Cy5 fluorescence emission when exciting PhycoE reports on the formation of Cy5-SA-PhycoE-biotin complexes that results from content mixing. To investigate whether there is asymmetry in these assays, we performed multiple sets of experiments that in each case involved preparation of four types of liposomes. Two types of liposomes contained the usual fluorescent probes as described above and will be generally referred to as V1 and T1 (for the synaptobrevin-containing and the syntaxin-1-SNAP-25-containing liposomes, respectively). In the other two types of liposomes we reversed the fluorescent probes, i.e. synaptobrevin-liposomes contained PhycoE-biotin (referred to as V2 liposomes) and syntaxin-1-SNAP-25 liposomes contained MB- and NBD-lipids, as well as Cy5-SA (referred to as T2) (Fig. 1A). The protein-to-lipid (P:L) ratio used to prepare the liposomes was 1:600 in all cases, and V and T liposomes were mixed at equal concentrations. These conditions differed from those of our standard reconstitutions, which are more asymmetric (the P:L ratios are 1:500 and 1:800 for V- and T-liposomes, respectively, and the concentration of T-liposomes is two-fold higher than that of V-liposomes to facilitate the possibility of more than one round of fusion^39^).

Liposome preparations were extensively characterized to ensure that any asymmetry observed in lipid and content mixing between V1-T1 and V2-T2 reactions did not arise from differences in the size of the liposomes, their protein content and the amounts of incorporated fluorescent probes (illustrated for representative liposomes samples in Fig. 1). Total lipid concentrations were measured by the Stewart method^41^ and used accordingly to dilute the liposomes to the desired concentrations to acquire fluorescence spectra and perform lipid and content mixing assays. Analyses by SDS-PAGE confirmed that comparable amounts of synaptobrevin were incorporated into V1 and V2 liposomes, and comparable amounts of syntaxin-1 and SNAP-25 were incorporated into T1 and T2 liposomes (Fig. 1B, Supporting Fig. S1). Dynamics light scattering was used to examine the liposomes size distributions (e.g. Fig. 1C) and showed that T1, V1, T2 and V2 liposomes had similar average radii (Fig. 1D). Fluorescence emission spectra acquired with excitation of the MB fluorescence (at 370 nm) or the NBD fluorescence (at 465 nm) in the absence and presence of detergent revealed strong FRET between the MB and NBD probes in the intact V1 and T2 liposomes, and showed that the liposomes contained similar amounts of both probes (Fig. 1E,F). The NBD fluorescence emission intensity decreased to some extent upon detergent addition for both V1 and T2 liposomes, which likely arises because the fluorescence of this probe is highly sensitive to the environment^42^. Such changes should be kept in mind when examining the amount of lipid mixing observed in reconstitution assays. Fluorescence emission spectra acquired with excitation at 488 and 645 nm showed that the amounts of PhycoE-biotin trapped in T1 and V2 liposomes or Cy5-SA trapped in T2 and V1 liposomes were comparable (Fig. 1G,H). Note that, in this case, detergent addition did not cause substantial changes in the spectra and that there was no possibility for FRET in these experiments because the liposomes were characterized separately. The overall concentrations of the fluorescent probes in the liposomes measured from UV and fluorescence spectra and comparisons with standard samples were approximately those expected, indicating that there was little loss of lipids during liposome preparation, that fluorescent lipids were fully incorporated, and that content mixing probes were efficiently trapped in the liposomes.

**Figure 2:**
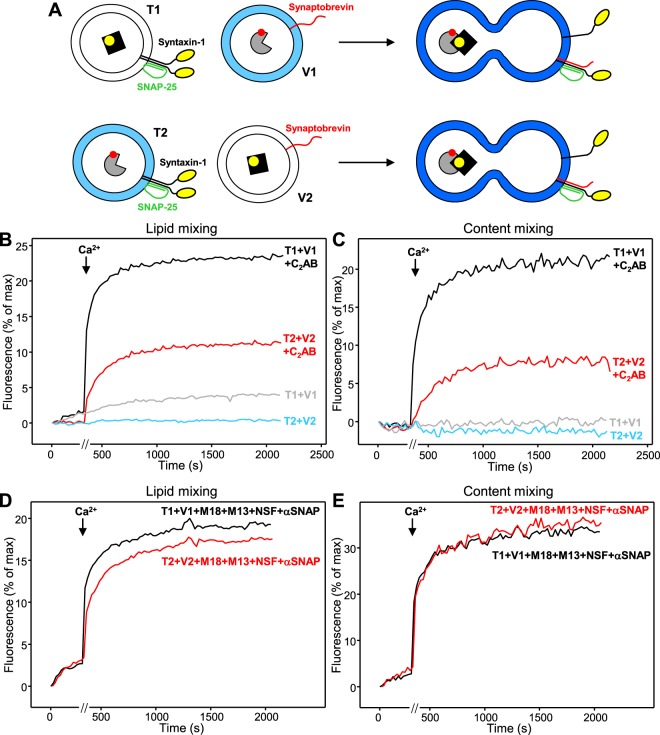
Example of asymmetry in lipid and content mixing between V and T liposomes in the presence of C_2_AB, but not in the presence of NSF, αSNAP, Munc18-1 and Munc13-1 C_1_C_2_BMUNC_2_C. (**A**) Diagrams illustrating the results expected from fusion reactions between T1 and V1 liposomes (above) or between T2 and V2 liposomes (below). The fluorescent probes used for lipid and content mixing are represented as in Fig. 1A. Upon fusion between a T and a V liposome, their bilayers merge and the ensuing dilution of the fluorescent lipids leads to de-quenching of the MB-PE fluorescence (represented in dark blue), while binding of Cy5-SA to PhycoE-Biotin due to content mixing leads to FRET between the probes and thus an increase in the emission fluorescence of Cy5-SA upon excitation of PhycoE-Biotin. (**B**–**E**) Lipid mixing (**B**,**D**) between V1 and T1 liposomes, or between V2 and T2 liposomes, was measured from the fluorescence de-quenching of Marina Blue-labeled lipids present in the V1 or T2 liposomes, and content mixing (**C**,**E**) was monitored from the development of FRET between PhycoE-Biotin trapped in the T1 or V2 liposomes and Cy5-SA trapped in the V1 or T2 liposomes. In (**B**,**C**), the assays were performed in the absence (gray and blue traces) or presence (black and red traces) of synaptotagmin-1 C_2_AB. In (**D**,**E**), the assays were performed in the presence of NSF, αSNAP, Munc18-1 (M18) and Munc13-1 C_1_C_2_BMUNC_2_C (M13). All experiments were started in the presence of 100 μM EGTA and 5 mM streptavidin, and Ca^2+^ (600 μM) was added after 300 s. Note that four reactions were performed in parallel and recording was stopped for about 60 s during the addition of Ca^2+^ to the four reactions [represented by a break (//) in the x axis]. The concentration of Cy5-SA used to prepare the V1 and T2 liposomes in all these experiments was 8 μM, and 4 μM PhycoE-biotin was used to prepare the T1 and V2 liposomes.

In multiple studies where we used simultaneous lipid and content mixing assays^8,15,20,22,27,43^, we observed some degree of variability in the activity of the liposomes that is natural in reconstitution experiments but needs to be considered when comparing results obtained with different liposome preparations. Thus, we performed multiple sets of experiments to verify that differences in lipid and/or content mixing observed in T1-V1 and T2-V2 reactions did not arise from such variability. We present two sets of experiments that illustrate the key conclusions that we could consistently draw from the overall data. Figure 2 shows results obtained in initial sets of experiments in which the concentration of Cy5-SA present during preparation of V1 and T2 liposomes was 8 μM and the concentration of PhycoE-biotin used to prepare T1 and V2 liposomes was 4 μM, which we commonly used in previous studies to prepare and V- and T-liposomes, respectively. We tested three conditions in these experiments: one where we mixed V1 with T1 or V2 with T2 liposomes alone; another where the liposomes were mixed in the presence of a fragment spanning the two C_2_ domains that form most of the cytoplasmic region of synaptotagmin-1 (C_2_AB), which was shown previously to strongly stimulate lipid and content mixing between V- and T-liposomes (e.g.^8,33,44^); and another in which the liposomes were mixed in the presence of the key factors that mediate SNARE complex assembly and disassembly, namely NSF, αSNAP, Munc18-1 and a fragment spanning the conserved C-terminal region containing the C_1_, C_2_B, MUN and C_2_C domains of Munc13-1 (C_1_C_2_BMUNC_2_C)^7,8,15^. As expected based on our previous studies (e.g.^8^), little or no lipid mixing or content mixing were observed between V1 and T1 or V2 and T2 liposomes alone, and synaptotagmin-1 C_2_AB strongly stimulated both lipid and content mixing between T1 and V1 liposomes in the presence of Ca^2+^ (Fig. 2B,C). However, Ca^2+^-dependent lipid and content mixing between V2 and T2 liposomes in the presence of C_2_AB was much less efficient (Fig. 2B,C), showing that there can be a striking asymmetry not only in lipid mixing but also in content mixing in liposome fusion reactions mediated by the neuronal SNAREs. In contrast, Ca^2+^-dependent lipid and content mixing were highly efficient for both the V1-T1 and the V2-T2 reactions in the presence of NSF, αSNAP, Munc18-1 and Munc13-1 C_1_C_2_BMUNC_2_C (Fig. 2D,E).

**Figure 3:**
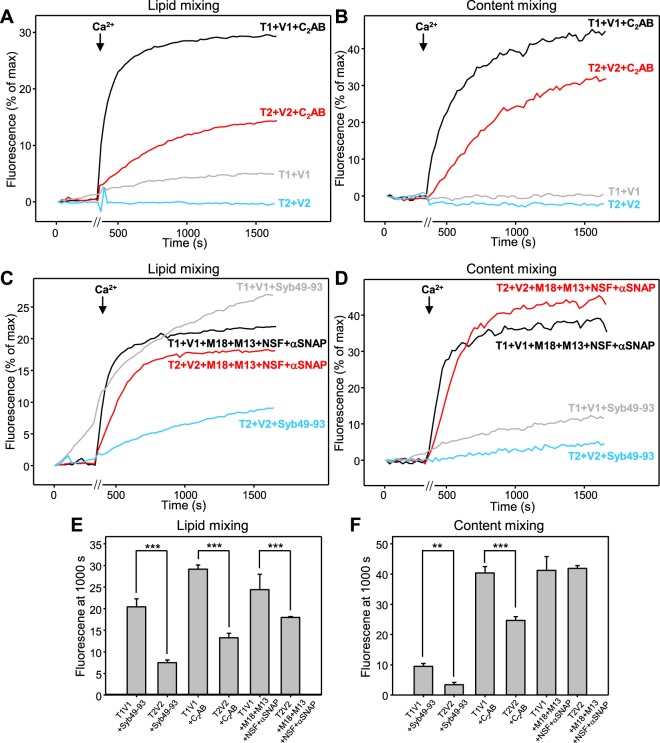
Example of asymmetry in lipid and content mixing between V and T liposomes in the presence of C_2_AB or Syb49-93, but not in the presence of NSF, αSNAP, Munc18-1 and Munc13-1 C_1_C_2_BMUNC_2_C. Lipid mixing (**A**,**C**) between V1 and T1 liposomes, or between V2 and T2 liposomes, was measured from the fluorescence de-quenching of Marina Blue-labeled lipids present in the V1 or T2 liposomes, and content mixing (**B**,**D**) was monitored from the development of FRET between PhycoE-Biotin trapped in the T1 or V2 liposomes and Cy5-SA trapped in the V1 or T2 liposomes. In (**A**,**B**), the assays were performed in the absence (gray and blue traces) or presence (black and red traces) of synaptotagmin-1 C_2_AB. In (**C**,**D**), the assays were performed in the presence of Syb49-93 (gray and blue traces) or of NSF, αSNAP, Munc18-1 (M18) and Munc13-1 C_1_C_2_BMUNC_2_C (M13) (black and red traces). All experiments were started in the presence of 100 μM EGTA and 5 mM streptavidin, and Ca^2+^ (600 μM) was added after 300 s. Note that four reactions were performed in parallel and recording was stopped for about 60 s during the addition of Ca^2+^ to the four reactions [represented by a break (//) in the x axis]. The concentration of Cy5-SA used to prepare the V1 and T2 liposomes in all these experiments was 4 μM, and 4 μM PhycoE-biotin was used to prepare the T1 and V2 liposomes. (**E**,**F**) Quantification of the lipid mixing (**E**) or content mixing (**F**) observed after 1000 s in reconstitution assays in the presence of Syb49-93, synaptotagmin-1 C_2_AB or NSF, αSNAP, Munc18-1 and Munc13-1 C_1_C_2_BMUNC_2_C. Bars represent averages of the normalized fluorescence intensities observed after 1000 s in experiments performed in triplicate. Error bars represent standard deviations. Statistical significance and P values were determined by one-way analysis of variance (ANOVA) with Holm-Sidak test (^**^P < 0.01; ^***^P < 0.001).

The concentration of Cy5-SA used for preparation of V-liposomes in our standard conditions (8 μM) is double than that of PhycoE-biotin in preparation of T-liposomes (4 μM) to facilitate the observation of more than one round of liposome fusion, as the V-liposomes bear higher P:L ratios than the T-liposomes^8^. Since in our studies of asymmetry in lipid and content mixing we used the same P:L ratios in all liposomes, which in principle hinders the possibility of two rounds of fusion, we performed additional experiments where the concentration of Cy5-SA used to prepare V1 and T2 liposomes was the same (4 μM) as the concentration of PhycoE-biotin used to prepare the V2 and T1 liposomes. With such liposome preparations, we again observed much more efficient lipid mixing between V1 and T1 liposomes than between T2 and V2 liposomes in the presence of C_2_AB (Fig. 3A, black and red traces), and there was also asymmetry in the amount of content mixing (Fig. 3B, black and red traces) (see Fig. 3E,F for quantification). The asymmetry in content mixing appeared to be less marked than in the experiments of Fig. 2B,C, suggesting that such asymmetry may be related at least in part to the stoichiometry of binding of Cy5-SA to PhycoE-biotin (see below). In experiments performed in the presence of NSF, αSNAP, Munc18-1 and Munc13-1 C_1_C_2_BMUNC_2_C, we again observed similar amounts of content mixing for reactions with T1-V1 and T2-V2 liposomes (Fig. 3D,F). The lipid mixing efficiency was somewhat smaller for T2-V2 than for T1-V1 reactions (Fig. 3C,E), but the difference was small and might arise from presence of a somewhat larger amount of NBD-labeled lipids on the T2 than on the V1 liposomes.

In this set of experiments we also tested whether lipid and content mixing could be stimulated by addition of a peptide spanning the C-terminal half of the synaptobrevin SNARE motif (Syb49-93), which facilitates SNARE complex assembly because syntaxin-1 and SNAP-25 form heterodimers with 2:1 stoichiometry and Syb49-93 displaces the second syntaxin-1 molecule, thus generating an acceptor complex that readily binds to synaptobrevin^15,45^. Indeed, Syb49-93 induced efficient lipid mixing between T1 and V1 liposomes, but content mixing was highly inefficient, and much less lipid and content mixing was observed between T2 and V2 liposomes (Fig. 3C–F). These results show again a striking asymmetry in lipid and content mixing assays performed with neuronal SNAREs and correlate with the results obtained with their homologues from yeast vacuoles^38^. It is also worth noting that the extent of lipid and content mixing generally correlated in the experiments performed under the various conditions, but not always (Figs. 2 and 3). Note that the difference in lipid and content mixing between T1 and V1 liposomes in the presence of Syb49-93 (Fig. 3C,D, gray traces) is particularly dramatic and provides another illustration that extensive lipid mixing can occur with little content mixing. Thus, the asymmetries observed in lipid and content mixing may be related to some extent, but there may also be distinct factors that contribute to asymmetry in lipid but not in content mixing, or vice versa (see discussion).

**Figure 4:**
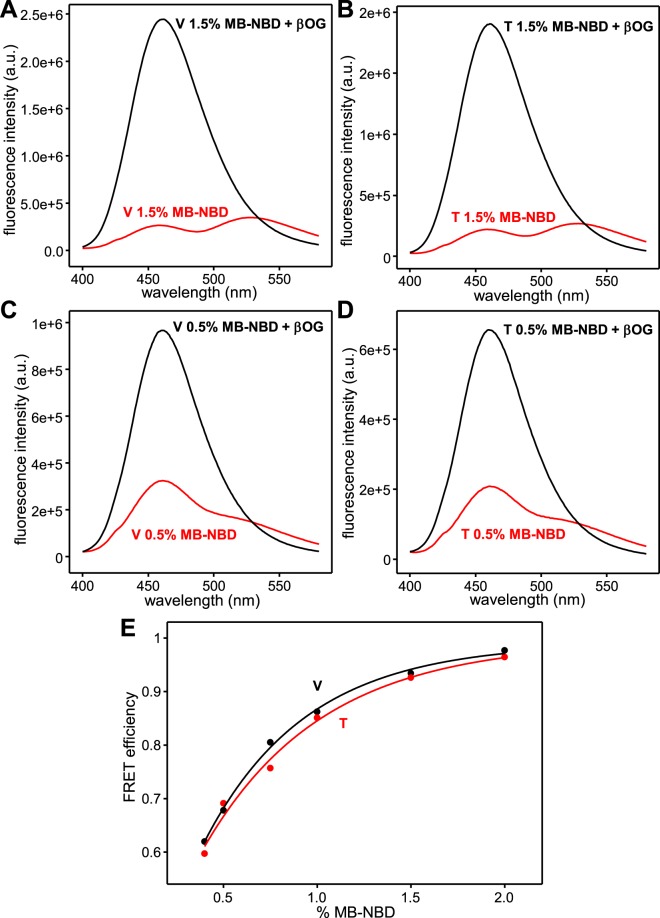
Comparable FRET between MB- and NBD-fluorescent lipids in V and T liposomes. (**A**–**D**) Fluorescence emission spectra of V (**A**,**C**) or T (**B**,**D**) liposomes containing 1.5% (**A**,**B**) or 0.5% (**C**,**D**) MB- and NBD-labeled lipids before (red traces) or after (black traces) addition of βOG. (**E**) The observed FRET efficiency was plotted against the percentage of MB- and NBD-fluorescent lipids in V (black dots) or T (red dots) liposomes. Curves show fits to the exponential function f = y0 + a * (1 − exp(−b * x)), where f is the FRET efficiency, x is the percentage of fluorescent lipids and the fitted parameters were: y0 = 21.9, a = 77.2 and b = 1.83 for V liposomes, and y0 = 27.3, a = 72.1 and b = 1.58 for T liposomes.

Since the juxtamembrane regions of the neuronal SNAREs are highly basic and MB- and NBD-labeled lipids are negatively charged, we considered the possibility that differences in the interactions of the SNAREs with the lipids might cause distinct FRET efficiencies between MB and NBD in the V1 and T2 liposomes, which could explain at least in part the observation of asymmetry in lipid mixing. To investigate this possibility, we prepared several samples of V- and T- liposomes with different percentages of MB- and NBD-labeled lipids. The fluorescence emission spectra that we obtained in the absence and presence of detergent for V- and T-liposomes with the same percentages of MB- and NBD-labeled lipids were very similar (e.g. Fig. 4A–D) and, correspondingly, comparable calibration curves were obtained when we quantitated the FRET efficiency for the different MB- and NBD-labeled lipid percentages (Fig. 4E). We note that substantial FRET efficiency (ca. 0.6) remained even for V- or T-liposomes with 0.5% MB- and NBD-labeled lipids, showing that multiple rounds of fusion with unlabeled liposomes are required to fully lose the FRET.

**Figure 5:**
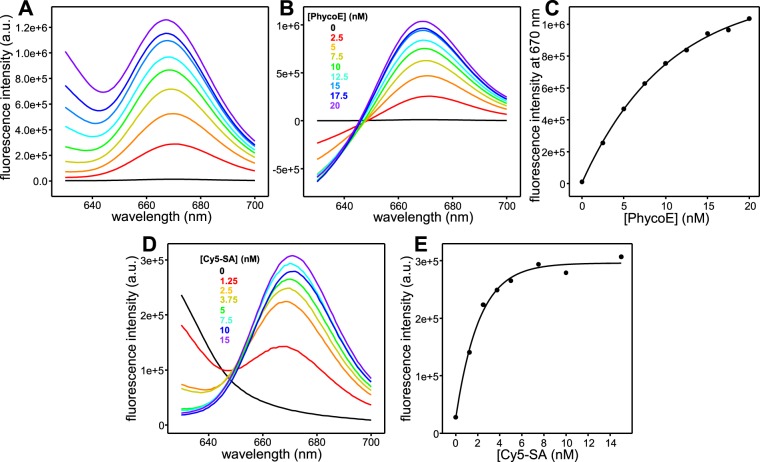
Analysis of FRET between Phyco-E biotin and Cy5-SA. (**A**) Fluorescence emission spectra acquired at the region of Cy5 fluorescence emission while exciting the Phyco-E fluorescence at 565 nm in samples containing 5 nM Cy5-SA and different concentrations of PhycoE-biotin as indicated by the color code in panel (B). (**B**) Corrected fluorescence emission spectra obtained after subtracting spectra acquired at different concentrations of PhycoE-biotin (without Cy5-SA) from the spectra shown in panel (A). (**C**) Plot of the corrected fluorescence intensity at 670 nm observed in the spectra of panel (B) as a function of PhycoE-biotin concentration. The data were fit to the exponential function f = y0 + a * (1 − exp(−b * x)), where f is the fluorescence intensity (a.u.), x is the PhycoE-biotin concentration (nM) and the fitted parameters were y0 = 6.27 * 10^3^, a = 1.19 * 10^6^, and b = 0.097. (**D**) Fluorescence emission spectra acquired at the region of Cy5 fluorescence emission while exciting the Phyco-E fluorescence at 565 nm in samples containing 5 nM PhycoE-biotin and different concentrations of Cy5-SA as indicated by the color code. (**E**) Plot of the corrected fluorescence intensity at 670 nm observed in the spectra of panel (D) as a function of Cy5-SA concentration. The data were fit to the exponential function f = y0 + a * (1 − exp(−b * x)), where f is the fluorescence intensity (a.u.), x is the Cy5-SA concentration (nM) and the fitted parameters were y0 = 2.66 * 10^4^, a = 2.69 * 10^4^, and b = 0.472.

An important aspect that might underlie asymmetry in content mixing is the stoichiometry of binding of Cy5-SA to PhycoE-biotin, which is unclear because SA is tetrameric^46^ and PhycoE is a hexamer of α/β subunits^47^. Thus, binding of multiple PhycoE-biotin hexamers to a Cy5-SA tetramer (or vice versa) and subsequent binding of Cy5-SA tetramers and PhycoE-biotin hemaxers might lead to formation of large polymeric complexes, but steric constraints might hinder growth of such polymers. To examine the stoichiometry of binding experimentally, we performed titrations of 5 nM Cy5-SA with different concentrations of PhycoE-biotin, exciting the PhycoE fluorescence and monitoring the development of Cy5 emission fluorescence by FRET. Spectra of 5 nM Cy5-SA in the absence of PhycoE-biotin showed negligible Cy5-SA emission, but addition of increasing amounts of PhycoE-biotin led to a progressively increased Cy5 fluorescence emission intensity (Fig. 5A). Note that the increase in fluorescence intensity at the maximum of the Cy5-SA emission spectrum (ca. 670 nm) arose in part because of the increasingly large tail of the very strong fluorescence emission from PhycoE-biotin as its concentration was increased (Fig. 5A). To account for the contributions from the tail, we acquired fluorescence emission spectra of different concentrations of PhycoE in the absence of Cy5-SA and subtracted these control spectra from those obtained in the presence of 5 nM Cy5-SA (Fig. 5B). Although the subtraction is not perfect because the fluorescence emission of PhycoE decreases in the spectra obtained in the presence of Cy5-SA due to FRET, plotting the corrected fluorescence intensity at 670 nm as a function of PhycoE-biotin concentration showed that Cy5-SA fluorescence increased almost linearly up to 10 nM PhycoE-biotin and did not saturate even at 20 nM PhycoE-biotin (Fig. 5C). The strong tail of the PhycoE-biotin fluorescence emission spectrum hindered measurement of reliable Cy5-SA fluorescence at higher PhycoE-biotin concentrations. In another set of experiments, we kept the PhycoE-biotin constant at 5 nM and titrated with Cy5-SA, again exciting the PhycoE fluorescence and monitoring the development of Cy5 emission fluorescence by FRET. The fluorescence intensity increased gradually with Cy5-SA concentration but saturated at ca. 5 nM Cy5-SA, and much of the intensity had already developed at 2.5 nM Cy5-SA (Fig. 5D,E). These results show that FRET occurs efficiently for Cy5-SA:PhycoE-biotin ratios of about 1:2 or lower, but higher ratios lead to only small increases in the Cy5-SA emission fluorescence that saturate at a ratio of about 1:1.

## Discussion

Fundamental questions still remain about the mechanisms of neurotransmitter release and its regulation despite the extensive body of knowledge that has been built in this field. Reconstitution assays that attempt to recapitulate the steps that lead to Ca^2+^-triggered synaptic vesicle fusion using proteoliposomes bearing fluorescent probes have made key contributions to our understanding of neurotransmitter release and are expected to keep making major advances. The study presented here reveals that there can be dramatic asymmetry in the results obtained when the fluorescent probes are switched between synaptobrevin- and syntaxin-1-SNAP-25-containing liposomes, showing that switching the fluorescent probes can help to characterize better the involvement of the two types of liposomes in fusion and/or in lipid exchange, and emphasizing the importance of using multiple parameters to better understand the nature of the reactions between two types of liposomes.

The asymmetry in lipid and/or content mixing observed under some conditions in our reconstitution experiments most likely arises at least to some extent from heterogeneity in the liposomes, as our extensive characterization (e.g. Fig. 1) showed that the average properties of V1 and T1 liposomes are similar to those of V2 and T2 liposomes, respectively, and that the incorporation of fluorescent dyes in V2 and T2 liposomes is comparable to those in T1 and V1 liposomes, respectively. Some degree of heterogeneity in the sizes of the liposomes and the distribution of proteins incorporated in them is unavoidable in reconstitution of proteoliposomes^48^ and may lead to uneven participation of liposomes in fusion reactions. For instance, liposomes with larger protein density are more likely to participate in more than one round of fusion, whereas those with less protein may not participate in any fusion reaction. Even liposomes with the same size and protein density may participate in a different number of fusion reactions merely because of the random nature of the encounters with other liposomes. Moreover, the content mixing dyes may not be distributed evenly in the liposomes because only a few molecules of Cy5-SA and PhycoE-biotin are expected to be trapped in each liposome^39,40^ and hence a natural stochastic variability in the number of trapped molecules is expected even for liposomes of the same size. Because of uneven content dye distribution and because the liposomes have a range of sizes (e.g. Fig. 1C), fusion between two liposomes can lead to different amounts of Cy5-SA fluorescence. These various factors might lead to less than optimal FRET signal corresponding to content mixing even if the majority of vesicles fuse at least once and they may also lead to less than optimal lipid mixing signal, but the lipid and content mixing signals may be differentially affected by these heterogeneities. For instance, the maximum Cy5-SA fluorescence signal reflecting content mixing in reactions including NSF, αSNAP, Munc18-1 and Munc13-1 C_1_C_2_BMUNC_2_C did not reach 100% (Figs. 2E and 3D), likely due at least in part to the factors mentioned above, but the maximum lipid mixing signal observed (ca. 20%; Figs. 2D and 3C) is close to that expected for quantitative fusion of all liposomes based on the calibration of FRET in liposomes with different percentages of MB- and NBD-labeled lipids (Fig. 4E).

How can heterogeneity in liposomes lead to asymmetry in lipid and/or content mixing? Again, it is important to keep in mind that different factors may explain the asymmetries observed in lipid mixing and content mixing. Note for instance that in the experiments of Fig. 2B,C content mixing between T2 and V2 liposomes in the presence of C_2_AB appeared to be more impaired than lipid mixing, compared to the results obtained for V1 and T1 liposomes, whereas the opposite was observed in the experiments of Fig. 3A,B. The differences between the two sets of experiments might arise in part because in the latter we lowered the amount of trapped Cy5-SA in the V1 and T2 liposomes. In any case, the content mixing between V2 and T2 liposomes in the presence of C_2_AB was less efficient than that observed between V1 and T1 liposomes in both sets of experiments. A potential explanation for this observation might be provided if synaptobrevin is unevenly distributed on V liposomes. For instance, in experiments with the same amount of trapped Cy5-SA and PhycoE-biotin (Fig. 3), if 50% of the V1 liposomes are more active than the rest and each of these active V1 liposomes fuses on average with two T1 liposomes, whereas the less active V1 liposomes do not fuse, the lumen of the fused liposomes would contain a Cy5-SA:PhycoE-biotin ratio of ca. 1:2. Conversely, the same events with V2 and T2 liposomes would lead to a Cy5-SA:PhycoE-biotin ratio of ca. 2:1 in the fused liposomes, which would yield a lower Cy5-SA emission fluorescence than a 1:2 ratio (Fig. 5). The asymmetry in content mixing observed in experiments where SNARE complex assembly was stimulated by Syb49-93 (Fig. 3D) could also be explained by this model. Conversely, the lack of asymmetry in content mixing in the presence of NSF, αSNAP, Munc18-1 and Munc13-1 C_1_C_2_BMUNC_2_C (Figs. 2E and 3D) might arise because fusion is likely to be highly efficient under these conditions even for V liposomes that have lower amounts of synaptobrevin such that all or most V liposomes fuse with one T liposome. Note in this context that Munc18-1 and Munc13-1 are critical for proper assembly of trans-SNARE complexes^15^ and help to prevent the formation of antiparallel complexes between synaptobrevin and syntaxin-1 that are expected to hinder fusion^49^, whereas synaptotagmin-1 C_2_AB stimulates fusion by simultaneously binding to two membranes^42,50^ but likely does not prevent formation of antiparallel complexes. Activation of the SNAREs by Syb49-93 is expected to stimulate trans-SNARE complex assembly in the proper orientation, but much less efficiently than Munc18-1 and Munc13-1 C_1_C_2_BMUNC_2_C^15^.

The model to account for asymmetry in content mixing described above does not explain the asymmetry in lipid mixing observed in the presence of synaptotagmin-1 C_2_AB (Figs. 2B and 3A) and particularly the dramatic asymmetry observed in the presence of Syb49-93 (Fig. 3C). The latter observation is reminiscent of the lipid mixing asymmetry observed in experiments with yeast vacuolar SNAREs^38^ that motivated this work. As proposed to explain the lipid mixing asymmetry observed with the yeast vacuolar SNAREs, a plausible explanation for the asymmetric lipid mixing observed in our experiments is that the abundant basic residues present in the syntaxin-1 juxtamembrane region and additional basic residues at the C-terminus of the SNAP-25 SNARE motifs can catalyze transfer of negatively charged lipids such as MB-PE and/or NBD-PE from the V liposomes to the T liposomes. Such lipid transfer may occur when transient formation of trans SNARE complexes brings the two membranes into close proximity but fails to induce fusion or hemifusion. This mechanism would result in de-quenching of MB fluorescence in V1-T1 reactions but not in V2-T2 reactions. Note that synaptobrevin also contains basic residues at the C-terminus of its SNARE motif and could thus catalyze lipid transfer in the opposite direction, but the basic residues are likely more distant from the membrane surface in this case and the total number of basic residues is smaller than that in the membrane-proximal sequences of syntaxin-1 and SNAP-25.

Further studies will be required to test the models proposed here to explain the observed asymmetry in lipid and content mixing, and better understand the reasons for asymmetry. For instance, mutating the basic residues of the syntaxin-1 juxtamembrane regions will reveal whether they are involved in asymmetric lipid mixing, and a more systematic analysis changing the ratio of Cy5-SA:PhycoE-biotin trapped in the liposomes will help to establish whether this ratio indeed influences the observation of asymmetry in content mixing. It is also plausible that the asymmetries observed in our assays arise in part from the fact that T-liposomes contain DAG and PIP_2_, whereas V-liposomes do not. Differences in association between V- and T- liposomes in the presence of synaptotagmin-1 C_2_AB might also contribute to asymmetry in lipid and content mixing. Although C_2_AB is expected to cluster V and T liposomes indiscriminately because they both contain negatively charged phospholipids^42^, there might be some preferential clustering among T liposomes because they containing PIP_2_. In this context, it will be interesting to study whether full-length synaptotagmin-1 anchored on the V-liposomes also stimulates lipid and/or content mixing in an asymmetric fashion. Regardless of these possibilities, our results and those obtained with yeast vacuolar SNAREs^38^ clearly show that formation of SNARE complexes alone does not necessarily lead to membrane fusion even when they bring membranes close enough to exchange lipids, and emphasize again that observation of lipid mixing cannot be used as demonstration of membrane fusion. Lipid mixing assays can still be used to obtain complementary information, for example providing an indication that trans-SNARE complexes form even if they do not cause membrane fusion. Our study also shows that performing experiments with the fluorescence probes switched between the two types of liposomes provides additional information on the reactions between liposomes and helps to detect unexpected behaviors that inform on sample heterogeneity. Clearly, monitoring multiple parameters helps to understand better these complex reactions between liposomes, thus increasing further the power of reconstitution approaches to study membrane fusion.

## Materials and Methods

### Recombinant proteins

Expression in *E*. *coli* BL21 (DE3) cells and purification of full-length rat syntaxin-1A, full-length rat SNAP-25A (C84S, C85S, C90S, C92S), full-length rat synaptobrevin, rat synaptobrevin 49-93 (Syb49-93), rat synaptotagmin-1 C_2_AB (131–421 C277A), full-length *Bos Taurus* αSNAP, full length rat Munc18-1, and a rat Munc13-1 fragment spanning the C_1_C_2_BMUNC_2_C region (529–1725 Δ1408–1452) were performed as described previously^7,8,39^. Full-length Chinese hamster NSF (a kind gift from Minglei Zhao) was expressed in *E*. *coli* BL21 (DE3) and purified as decribed^15,51^.

### Simultaneous lipid mixing and content mixing assays

Assays to simultaneously monitor lipid and content mixing between liposomes were performed as described^8,39^ with a few modifications to investigate whether there is asymmetry in lipid and/or content mixing when the fluorescent probes are switched. To prepare liposomes, 1-palmitoyl-2-oleoyl-sn-glycero-3-phosphocholine (POPC), 1,2-dioleoyl-sn-glycero-3-phospho-L-serine (DOPS), 1-palmitoyl-2-oleoyl-sn-glycero-3-phosphoethanolamine (POPE), L-a-Phosphatidylinositol-4,5-bisphosphate (PIP_2_), 1-palmitoyl-2-oleoyl-*sn*-glycerol (DAG), Marina Blue® 1,2-Dihexadecanoyl-sn-Glycero-3-Phosphoethanolamine (MB-PE), 1,2-dipalmitoyl-sn-glycero-3-phosphoethanolamine-N-(7-nitro-2-1,3-benzoxadiazol-4-yl) (NBD-PE) and cholesterol dissolved in chloroform were mixed at the desired ratios and then dried under a stream of nitrogen gas. The different types of liposomes had the following compositions: V1 liposomes, 39% POPC, 19% DOPS, 19% POPE, 20% cholesterol, 1.5% NBD-PE, and 1.5% MB-PE; V2 liposomes, 42% POPC, 19% DOPS, 19% POPE and 20% cholesterol; T1 liposomes, 38% POPC, 18% DOPS, 20% POPE, 20% cholesterol, 2% PIP2, and 2% DAG; and T2 liposomes, 35% POPC, 18% DOPS, 20% POPE, 20% cholesterol, 2% PIP2, 2% DAG, 1.5% NBD-PE and 1.5% MB-PE. Dried lipid mixtures were re-suspended in 25 mM HEPES pH 7.4, 150 KCl, 1 mM TCEP, 10% glycerol, 2% β-OG. Purified SNARE proteins and fluorescently labeled content mixing molecules were added to the lipid mixtures to make the syntaxin-1:SNAP25:lipid ratio 1:5:600 for T1 and T2 liposomes, and the synaptobrevin:lipid ratio 1:600 for V1 and V2 liposomes. V1 and T2 liposomes were prepared in the presence of 4 or 8 µM Cy5-SA, as indicated in the figure legends, while V2 and T1 liposomes were prepared in the presence of 4 µM R-Phycoerythrin-Biotin (PhycoE-biotin). The mixtures were incubated at room temperature and dialyzed against the reaction buffer (25 mM HEPES pH 7.4, 150 mM KCl, 0.5 mM TCEP, 10% glycerol) with 2 g/L Amberlite XAD-2 beads (Sigma) 3 times at 4 °C. Proteoliposomes were purified by floatation on a three-layer histodenz gradient (35%, 25%, and 0%) and harvested from the top layer. To simultaneously measure lipid mixing from de-quenching of Marina Blue lipids and content mixing from the development of FRET between PhycoE-Biotin and Cy5-SA, V1 liposomes (0.25 mM total lipid) were mixed with T1 liposomes (0.25 mM total lipid), or V2 liposomes (0.25 mM total lipid) were mixed with T2 liposomes (0.25 mM total lipid), in reaction buffer containing 0.1 mM EGTA (200 µL total volume). Some reactions were performed by mixing the liposomes in the presence of 2 μM synaptotagmin-1 C_2_AB or 10 μM Syb49-93, as indicated in the figure legends. For reactions that included Munc18-1, Munc13-1 C_1_C_2_BMUNC_2_C, NSF and αSNAP, T1 or T2 liposomes were first pre-incubated with 0.8 µM NSF, 2 µM αSNAP, 2.5 mM MgCl_2_, 2 mM ATP, 0.1 mM EGTA, 0.5 mM TCEP and 1 µM Munc18-1 at 37 °C for 25 minutes. They were then mixed with donor V-liposomes, 0.5 µM Munc13-1 C_1_C_2_BMUNC_2_C, and 1 µM excess SNAP25. All experiments were performed at 30 °C and 0.6 mM Ca^2+^ was added at 300 s. To monitor lipid and content mixing, the fluorescence signal from Marina Blue (excitation at 370 nm, emission at 465 nm) and Cy5 (excitation at 565 nm, emission at 670 nm) were recorded on a PTI Quantamaster 400 spectrofluorometer (T-format) equipped with a rapid Peltier temperature controlled four-position sample holder, which allowed performance of four reactions in parallel. At the end of the reactions, 1% β-OG was added to solubilize the liposomes and the lipid mixing data were normalized to the maximum fluorescence signal. Most experiments were performed in the presence of 5 µM streptavidin to ensure that the content mixing signal arises from liposome fusion rather than liposome leakiness, and control experiments without streptavidin were performed to measure the maximum Cy5-SA fluorescence after detergent addition for normalization of the content mixing data.

### Dynamic light scatting

DLS experiments were performed at 25 °C using a Wyatt Dynapro Nanostar (Wyatt Technology) instrument equipped with a temperature controlled microsampler as previously described^8^. Liposomes were diluted to the same concentration used for the lipid and content mixing assays (0.25 mM total lipid) in reaction buffer. Three independent measurements of particle size distribution, each consisting of the average of 10 data points, were performed for each sample.

### Fluorescence spectroscopy

Fluorescence spectra were acquired at 30 °C on a PTI Quantamaster 400 spectrofluorometer (T-format) equipped with a rapid Peltier temperature controlled four-position sample holder. All slits were set to 1.2 or 1.25 mm. Liposome samples were prepared as described above for the lipid and content mixing assays, except for samples used to calibrate FRET between MB-PE and NBD-PE (Fig. 4), which were prepared with the indicated percentages of these fluorescent lipids. Samples were diluted in buffer containing 25 mM HEPES pH 7.4, 150 mM KCl, 0.5 mM TCEP, 0.1 mM EGTA and 10% glycerol, and spectra were acquired with the excitation wavelength indicated in the corresponding figure legends. The FRET efficiency between MB-PE and NBD-PE at different percentages of fluorescent lipids (Fig. 4E) were calculated with the formula E = (F_0_ − F)/F_0_, where F and F_0_ are the MB-PE fluorescence emission intensities observed for a given liposome sample before and after addition of 1% βOG, respectively. F_0_ was corrected by the dilution associated with detergent addition.

## Supplementary information

Supplementary Figure 1.

## Data Availability

The datasets generated during and/or analyzed during the current study are available from the corresponding author on reasonable request.
