# Supplementary material for: Analysis of asymmetry in lipid and content mixing assays with reconstituted proteoliposomes containing the neuronal SNAREs
Source: Sci Rep. 2020 Feb 19;10:2907. doi: 10.1038/s41598-020-59740-7 (PMC7031292; doi:10.1038/s41598-020-59740-7)
Supplement: Supplementary file 1 — Supplementary Figure 1. [file 41598_2020_59740_MOESM1_ESM.pdf]

Supplementary Material for:

Analysis of asymmetry in lipid and content mixing assays with reconstituted proteoliposomes containing the neuronal SNAREs

Yun-Zu Pan[1,2,3], Xiaoxia Liu[4] and Josep Rizo[1,2,3*]

[1]Department of Biophysics, University of Texas Southwestern Medical Center, Dallas, Texas, United States; [2]Department of Biochemistry, University of Texas Southwestern Medical Center, Dallas, Texas, United States; [3]Department of Pharmacology, University of Texas Southwestern Medical Center, Dallas, Texas, United States; [4]Key Laboratory of Cell Differentiation and Apoptosis of Chinese Ministry of Education, Department of Pathophysiology, Shanghai Jiao Tong University School of Medicine, Shanghai, China.

[*]For correspondence: Jose.Rizo-Rey@UTSouthwestern.edu

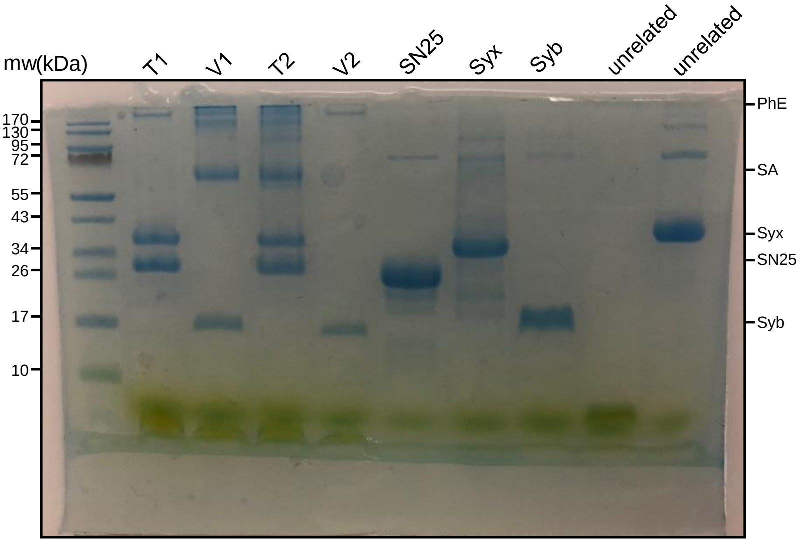

Supporting Figure S1. Uncropped image of the gel used to generate Fig. 1A. In addition to the lanes corresponding to the molecular weight markers and the T1, V1, T2 and V2 liposomes, there are three lanes corresponding to loading controls for SNAP-25 (SN25), syntaxin-1 (Syx) and synaptobrevin (Syb), as well as two lanes unrelated to this study. The positions of molecular weight markers are indicated on the left, and those of synaptobrevin (Syb), SNAP-25 (SN25), syntaxin-1 (Syx), SA and PhycoE (PhE) are indicated on the right.
